# Dosimetry during intramedullary nailing of the tibia: Patient and occupational exposure

**DOI:** 10.3109/17453670903350057

**Published:** 2009-10-01

**Authors:** George Kirousis, Harry Delis, Panagiotis Megas, Elias Lambiris, George Panayiotakis

**Affiliations:** ^1^Department of Orthopaedics, School of Medicine, University of PatrasPatrasGreece; ^2^Department of Medical Physics, School of Medicine, University of PatrasPatrasGreece

## Abstract

**Background** Intramedullary nailing under fluoroscopic guidance is a common operation. We studied the intraoperative radiation dose received by both the patient and the personnel.

**Patients and methods** 25 intramedullary nailing procedures of the tibia were studied. All patients suffered from tibial fractures and were treated using the Grosse-Kempf intramedullary nail, with free-hand technique for fixation of the distal screws, under fluoroscopic guidance. The exposure, at selected positions, was recorded using an ion chamber, while the dose area product (DAP) was measured with a DAP meter, attached to the tube head. Thermoluminescent dosimeters (TLDs) were used to derive the occupational dose to the personnel, and also to monitor the surface dose on the gonads of some of the patients.

**Results** The mean operation time was 101 (48–240) min, with a mean fluoroscopic time of 72 seconds and a mean DAP value of 75 cGy·cm^2^. The surface dose to the gonads of the patients was less than 8.8 mGy during any procedure, and thus cannot be considered to be a contraindication for the use of this technique. Occupational dose differed substantially between members of the operating personnel, the maximum dose recorded being to the operator of the fluoroscopic equipment (0.11 mSv).

**Interpretation** Our findings underscore the care required by the primary operator not to exceed the dose constraint of 10 mSv per year. The rest of the operating personnel, although they do not receive very high doses, should focus on the dose optimization of the technique.

## Introduction

During intramedullary nailing of long bones, intraoperative fluoroscopic guidance is required for the reduction of the fracture, the placement of the nail, and the fixation of distal screws. The use of radiation, combined with the relatively young age of the patients, highlights the need to measure the exposure of the patient and personnel and to find ways of optimizing the use of intraoperative fluoroscopy (Hofstetter et al. 1999, Kahler 2001).

In the past, several authors have measured the patient dose and the occupational dose during fluoroscopically guided operations using slow dosimetric films (Fitousi et al. 2006). Later, for the same measurement, thin-layer lithium fluoride thermoluminescence dosimetry (TLD) chips were used (Miller et al. 1983, Sanders et al. 1993), carrying the advantage of higher precision and minimum interference, due to their small sizes. In the last few years there has been increased interest in estimation of patient radiation exposure by means of dose area product (DAP). Due to its precision and independence from the projection and the technique used, it is considered the primary index to be used in order to establish diagnostic reference levels (DRLs) for fluoroscopic procedures (Carwley and Rogers 2000, Hart et al. 2004, Fitousi et al. 2006).

We measured radiation doses, both patient and occupational, associated with intramedullary nailing procedures. During all procedures, Grosse-Kempf intramedullary nailing was applied, using the free-hand technique for the fixation of the distal screws. The aim was to determine the dose to the employees who participated in the operation, and to examine whether the annual dose constraints (ICRP 2007) would be exceeded for a typical workload. In addition, through dose mapping of the operating room different positions of the personnel around the operation table were examined in order to study the possibility of minimizing the occupational dose. Regarding the safety of patients, our main aim was to quantify the amount of energy imparted to the gonads, since due to their positioning they would be close to the primary X-ray beam.

## Material and methods

The complete dosimetric study during intramedullary nailing, which was performed in a university hospital, originally included 27 patients. During 2 tibial nailing procedures severe complications occurred, resulting in increased fluoroscopy time. These patients were removed from the sample concerning patient dose, while their results were included in the occupational dose study. Thus, the final sample for the DAP estimation involved 25 patients, 10 of whom were also monitored with TLDs for the estimation of entrance surface dose (ESD).

All procedures were carried out under the fluoroscopic guidance of 2 C-arm devices of the same type (Philips BV 25 Libra/Gold). Both devices were under a complete quality-control program for consistency of performance, according to national and international protocols (Conference of Radiation Control Program Directors (CRCPD) 2001).

### Area exposure survey

In the first part of the study, and before in vivo measurements, the operation room was mapped in terms of dose equivalent per DAP. This would allow us to inform the medical staff participating in the operation about the positions of lowest exposure that they should ideally occupy. The patient set-up was simulated using blocks of perspex of the appropriate geometry and thickness (dimensions: 60 cm × 30 cm × 10 cm). The exposure was recorded under operation set-up using an area survey meter (Victoreen Ion Chamber 451P), while irradiating with typical fluoroscopic conditions for intramedullary nailing. At the same time, the DAP was recorded for a fixed field size at the phantom entrance plane.

For the purpose of area mapping, the room was divided into rectangular areas of 0.5 m × 0.5 m, at the center of which the dose was measured. The point of each measurement was positioned 1 m above the floor, which is the mean height of the position of the gonads—one of the most radiosensitive types of organ, according to the International Commission of Radiation Protection ICRP 2007.

### Occupational exposure

Following the ICRP recommendations, the occupational exposure was measured to assure that the annual dose constraint of 10 mSv was not exceeded, under normal circumstances. For this purpose, thermoluminescent dosimeters (TLD-100 LiF:Mg, Ti, Harshaw-Bicron, OH, USA) were used (in sets of 3 chips) on the medical personnel, during all intramedullary nailing procedures, without replacement or periodical readout. This was unavoidable since the threshold dose of the TLDs, in addition to the low doses imparted, does not allow their use per single operation. One more set was used for estimation of background radiation, and the net difference between the mean value of the 3 chips of each set and the background dose was considered to be the final measurement. For the physician operating the C-arm fluoroscopic system during the surgery, 2 sets of TLDs were used and the effective dose was derived according to the recommendations of the National Council on Radiation Protection and Measurements (NCRP 1995). The first set was placed at the neck above the lead apron and the second one was placed at waist level below the apron. The primary operator's whole-body dose was calculated according to the formula:

**Figure:**
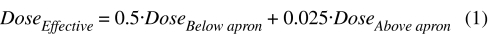


Although this methodology leads to an overestimation of up to 100%, it can be considered representative of the amount of energy imparted to the physician. Under normal circumstances, only the operator of the fluoroscopic equipment was inside the room during the irradiation, and thus he was the only member of staff wearing a protective apron on a regular basis. For the rest of the personnel, only 1 TLD set was used, which was placed at the neck, and so the whole-body dose was calculated by a similar formula for single dosimeter measurement (Padovani and Rodella 2001):

**Figure:**
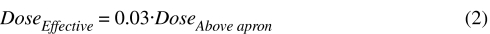


#### Patient dose

Measurement of dose to the patient during intramedullary nailing was an essential part of the study, in order to ensure that it was within an acceptable range and for establishment of local and national DRLs. During an intramedullary nailing procedure, the exposure parameters vary widely and the primary X-ray beam is directed at different areas and angles, in order to image different regions of interest. The DAP value was selected for measurement of the X-ray tube output because of its precision and independence from the projection and the technique used. A DAP meter (PTW Diamentor M-4) was attached to the head of the tube throughout each procedure, and at the end of each operation the total DAP value and the total fluoroscopy time were recorded.

Apart from measurement of the tube output, which is indicative of the total amount of radiation, it is important to estimate the dose to critical organs, which could possibly receive an elevated dose (as described by the ICRP (2007)). In the case of intramedullary nailing in particular, the critical organs that are closest to the primary X-ray beam are the gonads. This is one of the most radiosensitive parts of the human body, which means that the effective dose is substantially increased.

The patients who undergo this particular procedure are mostly young. In order to estimate the dose to the gonads, thermoluminescent dosimeters (TLD-100 LiF:Mg) were placed at this specific part of the patient's body, during 10 procedures. The TLD indications were obtained and then converted to dose using the TLD calibration curve. Taking the background dose value into account, which was subtracted from each TLD measurement, the actual surface dose at the gonads of the patient was determined.

### Results and discussion

The total procedure time during the 25 tibial nailing procedures was 101 (SD 54) min, and ranged from 48 to 240 min. The corresponding fluoroscopy time was 71 (SD 40) seconds, ranging from 19 to 141 seconds. The fact that this time was shorter than in previously published studies (Table 1) can be attributed to different protocols being used in different hospital departments and perhaps also to the increased familiarization of the staff with this technique in our particular university hospital.

**Table 1.** Fluoroscopy time reported in various studies

	Mean (SD) fluoroscopy time, min	Experience of primary surgeon
Muller et al. 1998 (T)	4.16	
Madan and Blakeway 2002 (T)	0.56	Senior
	1.28	Middle grade
Blattert et al. 2004 (T + F)	4.43	Senior
	6.95	Junior
Hafez et al. 2005 (T + F)	2.58 (0.57)	
Malek et al. 2007 (T)	2.1 (1.3)	
Tsalafoutas et al. 2008 (T)	5.7 (3.5)	
Current study (T)	1.19 (0.67)	
T: tibial; F: femoral.		

**Figure 1.**
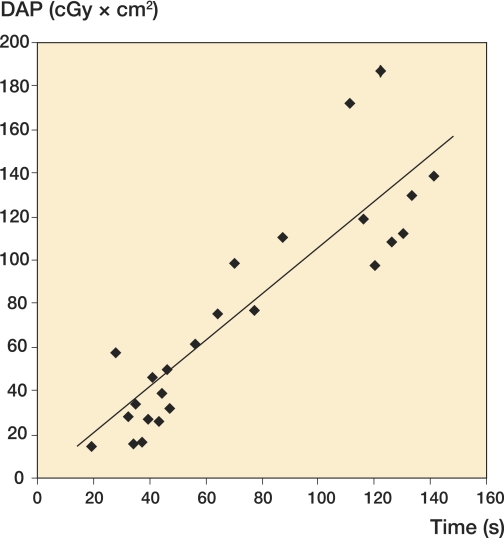
Dose area product (DAP) as a function of the total fluoroscopy time.

The DAP measurements were correlated to the fluoroscopy time of each operation and the results are shown graphically in Figure 1; they show a linear dependency. The DAP value was measured to be 75 (SD 50) cGy·cm^2^, ranging from 15.4 to 187.2 cGy·cm^2^. If a local DRL was to be set, based on these measurements, this would correspond to the third quartile of the DRL distribution, which in our case is 111 cGy·cm^2^. This is lower than the third quartile of 447 cGy·cm^2^ reported by Carwley and Rogers (2000), although the latter value corresponded to both tibial and femoral intramedullary nailing procedures and not only to the tibial procedures.

### Area exposure survey

**Figure 2.**
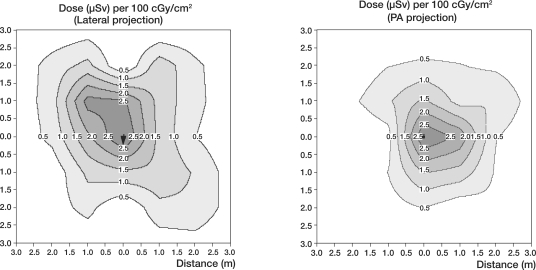
Ground plan of the operation room, with the corresponding iso-dose curves (in μSv) at a height of 1 m, for the lateral view (left panel) and posterior-anterior (PA) projection (right panel). The arrow and the cross show the tube direction and position, respectively.

The results of an area survey can prove to be useful for the operating personnel in order to select certain “low-dose” positions to stand during a procedure, thus minimizing the dose received and contributing to optimization of protection against radiation. For this reason, it was found useful to provide a graphical representation of the dose inside the operating room, using iso-dose curves and making use of 2 different projections (Figure 2).

From this figure, one can determine the dose rate at any selected position during fluoroscopy, taking into account that these curves indicate the dose with respect to the output of the fluoroscopic unit (expressed in terms of DAP values). In real clinical practice the dose to the personnel, as derived from the iso-dose curves, is significantly overestimated since whenever possible the personnel—with the exception of the operator of the fluoroscopic equipment—move out of the operation room during the irradiation and stand at the entrance to the operating theater, where the exposure rate was found not to exceed 0.7 μSv/h.

### Occupational exposure

The TLD measurements were used to derive the effective dose to the personnel, in order to know the level of risk and to estimate the number of operations that can be performed annually by the members of staff, without exceeding the proposed dose constraint of 10 mSv.

From the results obtained (Table 2), although they represent a high conservative estimate of the real effective dose, one can conclude that the maximum dose is imparted to the operator of the fluoroscopic equipment, as he/she is the only member of staff who has to remain close to the fluoroscopy unit during the entire irradiation time.

**Table 2.** TLD results and effective dose for the members of the operating team

TLD position	Mean TLD dose (mGy)	Effective dose per operation (mSv)
Primary surgeon	1.18	0.035
Assistant surgeon	0.74	0.022
Nurse	0.00	0.000
Equipment operator		
outside apron	1.02	0.105
inside apron	0.16	

Although, as already mentioned, these results are substantial overestimates, we suggest that the operator of the fluoroscopic equipment should not perform more than 95 operations annually, in this position. The primary surgeon can perform 282 operations annually, while the corresponding number for the assistant surgeon is 450. There is no need to set a limit for the nursing personnel, since the corresponding dose is very low.

### Patient dose

**Figure 3.**
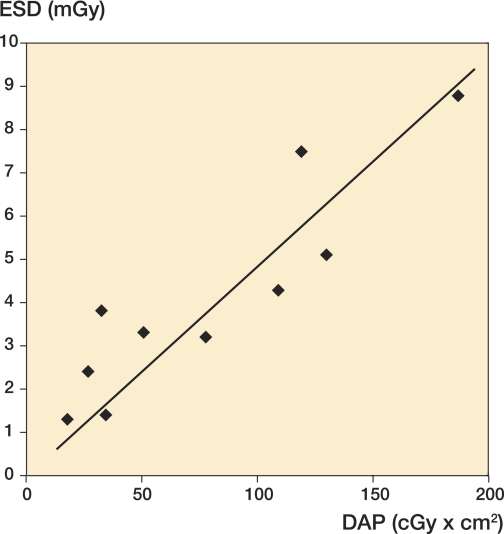
Measured patient entrance surface dose versus DAP values.

The measured surface dose to the gonads of the patients was found to range between 1.4 and 8.8 mGy (mean surface dose: 4.1 (SD 2.5) mGy). The expected linear correlation between the DAP value and the entrance surface dose to the gonads of the patient is shown in Figure 3. It is important to emphasize that this dose must not be confused with the maximum surface dose, which is much higher in the case of interventional procedures and can be recorded at the entrance point of the primary radiation.

The risk of hereditary effects, associated with fluoroscopy, can be estimated by multiplying the mean dose to the gonads by the hereditary effect risk factor of 0.2 × 10^-5^ mSv^-1^], as recommended by the ICRP 2007 for the population. If instead of the mean dose, which requires phantom measurements, the mean ESD from intramedullary nailing (4.1 mGy) is used, then the overestimated risk of hereditary effects can be calculated as follows:

**Figure:**
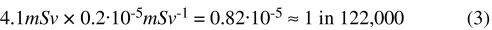


Making an extreme overestimation and considering that this mean surface dose is the actual dose imparted to the gonads, the skin, the bladder, and the colon (effective dose ∼ 1 mSv), the calculated cancer risk resulting from the operation would be less than 1 in 18,000.

It is important to note that even this overestimated result is extremely low. Taking into account that the technique is well justified and that its necessity is obvious, it can be concluded that the patient dose cannot be considered to be a contraindication for the use of this particular technique.

In summary, we found that medical personnel may attend a quite high number of these procedures annually without exceeding the dose constraints. Although this number easily covers the expected annual workload, it can be increased further if all the advice on radiation protection provided by the medical physicist concerning the use of protective aprons and standing positions is followed. As far as the patient is concerned, the dose to the gonads was found to be negligible.
